# Individual or Common Good? Voluntary Data Sharing to Inform Disease Surveillance Systems in Food Animals

**DOI:** 10.3389/fvets.2019.00194

**Published:** 2019-06-21

**Authors:** Andres M. Perez, Daniel C. L. Linhares, Andreia G. Arruda, Kimberly VanderWaal, Gustavo Machado, Carles Vilalta, Juan M. Sanhueza, Jerry Torrison, Montserrat Torremorell, Cesar A. Corzo

**Affiliations:** ^1^College of Veterinary Medicine, University of Minnesota, Minneapolis, MN, United States; ^2^College of Veterinary Medicine, Iowa State University, Ames, IA, United States; ^3^College of Veterinary Medicine, The Ohio State University, Columbus, OH, United States; ^4^College of Veterinary Medicine, North Carolina State University, Raleigh, NC, United States

**Keywords:** porcine reproductive and respiratory syndrome, epidemiology, surveillance, data sharing, US

## Abstract

Livestock producers have traditionally been reluctant to share information related to their business, including data on health status of their animals, which, sometimes, has impaired the ability to implement surveillance programs. However, during the last decade, swine producers in the United States (US) and other countries have voluntarily begun to share data for the control and elimination of specific infectious diseases, such as the porcine reproductive and respiratory syndrome virus (PRRSv). Those surveillance programs have played a pivotal role in bringing producers and veterinarians together for the benefit of the industry. Examples of situations in which producers have decided to voluntarily share data for extended periods of time to support applied research and, ultimately, disease control in the absence of a regulatory framework have rarely been documented in the peer-reviewed literature. Here, we provide evidence of a national program for voluntary sharing of disease status data that has helped the implementation of surveillance activities that, ultimately, allowed the generation of critically important scientific information to better support disease control activities. Altogether, this effort has supported, and is supporting, the design and implementation of prevention and control approaches for the most economically devastating swine disease affecting the US. The program, which has been voluntarily sustained and supported over an extended period of time by the swine industry in the absence of any regulatory framework and that includes data on approximately 50% of the sow population in the US, represents a unique example of a livestock industry self-organized surveillance program to generate scientific-driven solutions for emerging swine health issues in North America.

## Introduction

Although porcine reproductive and respiratory syndrome (PRRS) is one of the most important economic constraints to pork production in the US (1, 2), reporting of PRRS outbreaks is not mandatory in the country. In the absence of a regulatory framework, PRRS control and elimination actions are voluntary. At the field level, producers and veterinarians make decisions that seek to maximize profit while keeping the necessary standards for animal health and welfare. However, individual-level decisions may lead to complex and diverse epidemiological scenarios at a regional level. Because of the epidemiological features of PRRS virus (PRRSv) transmission, such as high levels of disease incidence, high variability and rapid mutation of the virus, intensiveness of production, and, in some cases, vertical integration of the industry, and limitations of current preventive and control methods, there is not much hope for disease control if programs are not simultaneously implemented at local (i.e., production system level) and regional levels (i.e., state or county levels) (3–6).

The perception that regional approaches are required to control the disease has led to the implementation of at least 30 voluntary regional PRRSv control or elimination projects in the US and Canada (7). None have succeeded in eliminating PRRSv regionally, and, arguably, most seem to have had limited success on significantly controlling the disease. Part of the limited progress on those regional projects may be attributed to inconsistencies in regional biosecurity compliance and suboptimal biosecurity of swine operations, incomplete regional producer participation, poor standardization, and availability of information on pathogen monitoring and surveillance systems, including lags in detection and communication about outbreaks (7). Lack of progress of regional control and eradication projects may also be at least in part associated with limited funding for disease and insufficient coordination of control activities. In contrast, one may argue that it is unknown what the epidemiological situation of the disease would be in the absence of those voluntary programs, and thus, the effectiveness of their implementation is debatable and subject to speculation.

Nevertheless, veterinarians and producers from individual farms and production companies, generically referred to as “production systems,” still need to make decisions intended to maximize their results (8) using the information available to them, which may lead to scenarios that are not compatible with disease control at a regional level. The tension between “individual” and “common” good leads to a complex relation of behaviors and attitudes, resembling a “game.” In this “game,” certain “players” (production systems) make decisions that may be conditional to the decisions made by other “players,” which, in turn, may result in a change of decisions taken by other “players.” This “game,” which in social economics corresponds to a concept referred to as “game theory” (9, 10), soon becomes dynamic, and the conditions required to control the disease at a regional level become, at minimum, difficult to reach. Thus, PRRSv control at the regional level becomes challenging, and often depends on regional leadership. If short- and long-term values of participation are not quantified and clear, it is unlikely that progress will be made.

Many stakeholders have perceived the social nature of PRRSv control at the regional level in the US. Spontaneous initiatives intended to voluntarily share knowledge have emerged in the country with the objective of promoting a greater good, i.e., control of a disease that affects the industry as a whole, even if such sharing may represent a potential risk or loss to their individual interest. Here, we review the largest voluntary initiative for data sharing among US swine producers, including a summary of its design and governance, and highlighting a number of epidemiological features of the disease that the project helped to elucidate over the last 10 years. The ultimate objective of this voluntary program is to build the capacity to respond in the event of an emerging disease, while supporting the prevention and control of endemic diseases of swine, such as PRRSv.

## PRRS and the Morrison's Swine Health Monitoring Project (MSHMP)

Since the late 1980's, PRRSv has been consistently generating losses in the US swine industry. Thanks to an effective collaboration between researchers, swine veterinarians, and swine producers, epidemiological characteristics of the disease were uncovered and preventive and control measures were implemented. Despite important improvements from a bioexclusion, biomanagement, and biocontainment standpoint, the virus continues to persist in US swine herds. Because there has historically been no documentation of disease occurrence metrics through space and time, the industry could not systematically assess whether the current situation was better or worse compared to previous years, generating uncertainty and speculation. Based on this knowledge gap and the need to further understand the epidemiology of the disease at larger spatial and temporal scales, a group of producers and practitioners decided to voluntarily share breeding herd PRRSv status for their respective production systems. Through weekly reporting, practitioners updated their respective PRRSv breeding herds' status, making the estimation of weekly cumulative incidence reports possible. The product of this effort was then shared back to participants in the form of weekly reports. Reports included information on disease prevalence, disease incidence, and proportion of herds in each PRRSv status. Reports also included benchmarking comparing numbers of participants to aggregated results from other participants in the database. The program's name changed through time and it is currently referred to as the Morrison's Swine Health Monitoring Project (MSHMP), in recognition of the late Dr. Robert Morrison, who was the driving force leading the inception and organization of the program. Compared to regional control projects, the MSHMP is larger, including information from reproduction farms (sow farms, multipliers, genetic nuclei) in a number of regions, whereas regional control projects are typically smaller, limited to a geographical area, and including also information nurseries, growers, and finishers.

**Figure 1:**
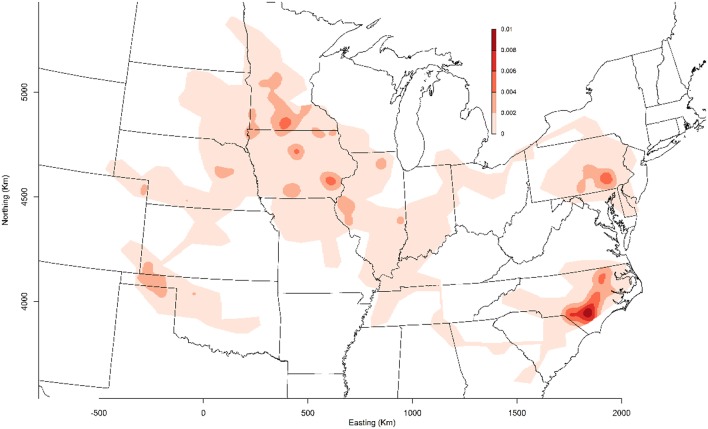
Geographical distribution of farms enrolled in the Morrison Swine Health Monitoring Project (MSHMP) as of February 2019.

MSHMP participants have agreed that PRRSv incidence graphs generated by their voluntary collaboration would be shared with the industry for benchmarking, disease monitoring, and promoting participation. In 2011, the project included 13 production systems that provided data related to PRRSv breeding herd status, location, and PRRSv interventions on a regular basis. Those systems represented approximately one million sows, which at the time, accounted for roughly 17% of the total US breeding herd based on USDA estimates (11). In 2013, porcine epidemic diarrhea virus (PEDv) and porcine delta-coronavirus (PDCoV) emerged in the US swine population (12, 13). This dramatic and dynamic situation provided an opportunity for producers to continue working together, and therefore PEDv and PDCoV were added to the list of diseases being monitored and reported on a voluntary basis (14). Over the years, others were prompted to join the project, which ultimately increased the representativeness of the database and thus provided a more accurate benchmark for the industry. Senecavirus A and pathogens associated with central nervous system disease were later added to the list of pathogens reported to MSHMP. At the time when this manuscript was written in February 2019, 38 production systems, accounting for ~50% of the US sow population, continue to provide their data for the benefit of the industry (Figure 1).

## MSHMP Project Design

Briefly, after each participant has voluntarily agreed to participate, a participation form and a data privacy agreement are signed prior to data acquisition. Then, farm-level information, such as location, herd size, farm type (e.g., farrow-to-wean, farrow-to-feeder, farrow-to-finish), genetic level (e.g., multiplier or commercial herd) and air filtration status is provided by each participant and stored in a central database housed at the University of Minnesota. One person within each participating system serves as point of contact for the project, becoming responsible for data sharing in direct communication with the MSHMP's data coordinator. Each week, participants share the list of sow farms that have changed their status, either worsening (e.g., outbreaks) or improving (e.g., ceased shedding or completed elimination). Once the information is received by the MSHMP data coordinator, it is reviewed for quality control and entered into the main database. Data are then used to estimate measures of disease occurrence such as incidence and prevalence. For incidence, a graph featuring the weekly and yearly cumulative incidence is presented. Incidence data are also used to create an exponentially weighted moving average graph, in which the magnitude and duration of the outbreak is graphed through time. Additionally, that figure depicts a threshold level (upper confidence limit of the two lowest seasons in the previous years) that marks the start and end of epidemic periods. The prevalence graph for PRRSv is based on a classification from the American Association of Swine Veterinarians (AASV), in which each PRRSv sow herd status is defined (15). The MSHMP report is comprised of 6 pages including participant logos and supporting/funding sources (page 1), the aggregate incidence and prevalence graphs for PRRSv (page 2) and PEDv (page 3), the Seneca Valley Virus and Atypical central nervous system case counts (page 4), a space for sharing the latest developments in swine related research referred to as “science page” (page 5), and the names and affiliations of all the individuals that receive the public report (page 6). Additional pages are shared with the project participants referring only to their own systems, and including incidence and prevalence graphs for both PRRSv and PEDv.

## MSHMP's Contribution to Swine Health Science

Since its inception in 2011, the MSHMP has played a critical role in providing data that scientists translated into science-driven solutions to help the US swine industry mitigating PRRSv impact. Here, we summarize some important swine disease features that the MSHMP has helped to elucidate and that promoted engagement and participation among producers.

### PRRSv Annual Occurrence Is Nationally Consistent but Regionally Different

For the last 10 years (2008–2018), PRRSv has maintained stable incidence levels on an annual basis, with an increase in the number of outbreaks, colloquially referred to as “PRRS season,” consistently starting between mid-October to mid-November (16). The only period that showed a substantial different incidence, compared to other periods, was in 2013–2014, when PEDv was first detected in the US (17).

However, seasonal dynamics seemed to differ across different states, with Minnesota, North Carolina, and Nebraska having more consistent seasonality than Iowa, and Illinois. Furthermore, there seems to be a secular pattern in the southern and southeastern regions of the country, with large epidemics occurring every 2–4 years (18, 19).

### PRRSv Impact May Be Mitigated by Implementing Certain Management Strategies

MSHMP investigators developed herd-level metrics of success of PRRSv control and elimination programs, such as time-to-stability (TTS), time to baseline productivity (TTBP), and total loss per thousand sows. Those metrics were used to compare the effect of multiple aspects or interventions on breeding herds affected with PRRSv. PRRSv-infected breeding herds achieved stability (i.e., producing PRRSv-negative piglets) significantly sooner, compared to other strategies, when they had reported a prior infection (i.e., existing partial herd immunity) and implemented herd closure (i.e., temporary interruption of replacement breeding stock introduction), and/or used wild type live virus inoculation as part of load-close-expose programs. Specifically, TTS was 7 weeks sooner for breeding herds adopting live-virus inoculation (LVI) as part of a load-close-expose program compared to those that used MLV vaccination protocols. Conversely, herds using MLV achieved TTBP 7 weeks sooner, and lost 1,443 piglets/1,000 sows less than herds using LVI. Altogether, economic analysis revealed an advantage for herds using MLV compared to LVI (20, 21).

More recently, MSHMP data have been used to estimate the breeding herd PRRS time-to-stability of a subset of breeding herds from six production systems that used similar testing criteria. A total of 82 breeding herds in the Midwestern US participated in this study, which accounted for ~250,000 sows. These herds reported 161 PRRS outbreaks between 2011 and 2017. Breeding herds that had PRRSv outbreaks during the spring and summer had a significantly longer TTS than herds that had outbreaks during the fall and winter. In addition, there was a significant difference between TTS between production companies suggesting that there are particular factors within production systems that may drive viral persistence in breeding herds (22).

### The Annual Cost of PRRSv to the Swine Industry has Decreased Since 2011

The MSHMP has been used to provide incidence data that helped to estimate the cost of PRRS, as part of various projects aimed at monitoring PRRS impact over time. Those data have helped in part to demonstrate that the annual cost of PRRS to the US swine industry has decreased $83.3 million from October 2010 ($663.91 million) to October 2016 ($580.62 million) (23), partially possibly due to increased number of breeding herds constantly immunizing sows with attenuated PRRS virus vaccines (Figure 1), which has been associated with reduced production losses (20).

**Figure 2:**
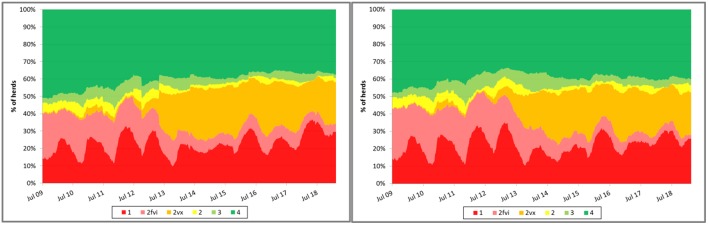
Evolution of porcine reproductive and respiratory syndrome (PRRS) status recorded in the Morrison Swine Health Monitoring Project (MSHMP) between 2009 and 2017 (left) and in 12 systems that consistently reported the status during the same time period (right). Breeding herds have been classified using a slightly modified terminology to the one proposed by Holtkamp et al. (15) that incorporates breeding herd shedding and immune status to increase its relevance to industry practices. Category 1 (red): positive unstable breeding herds that are undergoing a PRRSv outbreak and are weaning positive piglets. Category 2fvi (pink): herds that continue to expose breeding replacements to a live field virus strain (e.g., live-virus inoculation). Category 2vx (orange): herds that continue to expose breeding replacements or sows to PRRSv through the use of a modified-live PRRSv vaccine. Category 2 (yellow): herds that have uncertain PRRSv shedding status and positive PRRSv exposure status (animals are seropositive) with no clinical signs, no evidence of weanling piglet viremia and have stopped gilt and sow exposure to live virus. Category 3 (light green): herds that have negative PRRSv shedding status and have introduced breeding replacements that maintain seronegative status for more than 60 days. Category 4 (dark green): PRRSv-naïve herds in which pigs are negative for both shedding and exposure status for at least a year after reaching Category 3.

Some have suggested that, coincidently with the dissemination of research results demonstrating the impact of control measures on TTS, the proportion of sow farms in the MSHMP that have implemented vaccine-based control strategies substantially increased, suggesting that producers may have seen some value in the adoption and implementation of those research findings in the field (Figure 2). Noteworthy, however, it is also possible that the shift may be explained, at least in part, by other factors that were not formally assessed such as, for example, PED emergence in the country. In any case, data provided by participants has allowed MSHMP to compile and visualize the rate at which different control strategies have been adopted by participants.

### Environmental Factors Are Associated With the Odds of PRRS Outbreaks

Swine farm density has long been recognized as an important indicator for the risk of farms becoming infected with PRRSv and PEDv, even though the relative contribution of different routes of infection remain debated among swine practitioners and producers. Through the use of historical MSHMP data along with publicly available datasets, insights on environment-related factors that could affect the risk of PRRSv outbreaks were assessed. Swine sites located in regions with higher land slopes, and swine sites surrounded by trees and herbaceous coverage were protected from reporting a PRRSv outbreak compared to sites located in regions with lower land slopes and regions characterized by cultivated areas, respectively (24). However, the effects of slope may not be consistent across all regions, and may depend on the specific topography of the area (25). Precipitation, temperature, and land cover were all contributors for PRRSv spatial risk, with specific contribution amounts varying according to “subregion” in the US (26). For PEDv, temperature, wind speed, and vegetation have been identified as important modulators of risk, even in swine-dense areas (25). Taken together, these results demonstrate that, even for highly intensive pig production, environmental factors play an important role in determining outbreak risk and drive further disease spread.

### The Highly Infectious Nature of Swine Viral Infections, Along With the Strong Association With Epidemiological Factors May Facilitate Forecasting Disease Risk in Breeding Herds

For the past several decades, epidemiological modeling has been an important tool for understanding and predicting the spread of infectious diseases. The availability of farm-level outbreak data through MSHMP, combined with data on animal movements between farms (27), has enabled some of the first data-informed epidemiological models in the US swine industry. The availability of PRRSv genetic sequences from farms that became infected [see for example, (28)] allowed for the model to be fit to the observed spatiotemporal dynamics of the unfolding PED epidemic in 2013 (29). Data on which and when farms become infected also has opened the possibility of using this rich database to develop predictive models that could be used to forecast when a farm is expected to be at high risk. The potential for forecasting was initially explored using PED incidence data (25). Animal movement data available within the MSHMP was combined with environmental risk factors within a 10 km radius around breeding farms to identify major drivers of PED outbreaks, which included the total numbers of pig movements into neighboring farms, regional hog density, environmental, and weather factors such as vegetation, wind speed, temperature, and precipitation, and topographical features such as slope. Results suggest that PED occurrence may be predictable with an acceptable (i.e., >80%) level of accuracy, which eventually may lead to the design and implementation of a near real-time forecasting system for these diseases.

## Conclusions and Perspective

The US swine industry continues to reach important production levels together with expanding their breeding and growing herds. Infectious diseases are one of the most important limiting factors as they deteriorate performance and increases production costs. Furthermore, foreign animal diseases are of concern for the industry as they could potentially affect the industry's export market. Therefore, the US swine industry continues to work closely with researchers to seek answers and implement procedures to mitigate the burden of endemic diseases, while building capacity to respond against emerging and foreign animal diseases. A program like MSHMP, which its foundation is trust and voluntary participation, has had an important impact on the surveillance of pathogens for the national industry. An academic institution-led program has motivated producer and veterinarian collaboration for the greater good of the industry. Reasons why producers and veterinarians have been willing to share their data are difficult t to assess. Initially, there was an intention to standardize data collection and sharing, to improve situational awareness and facilitate the decision-making process considering the epidemiological situation of the disease in near real time and in the entire region, rather than on their own farms and systems only. However, motivation declines with time, and it is important to demonstrate the added value of the effort. In that regards, even though shared data are private and managed only by the MSHMP team, producers and veterinarians still obtain a benefit because important outputs, that help inform their decisions, are routinely shared with them. PRRSv has been used as a way to bring the industry together and build the methodology to create a program that has the potential to be adaptable to other diseases. MSHMP has demonstrated over the years that voluntary organization of swine producers and practitioners toward a common goal resulted in a powerful initiative that outweighed initial concerns about own data protection. Most importantly, such voluntary collaboration has also led to collaborative research involving a number of higher education institutions in the US that helped to elucidate some of the most important epidemiological features of endemic swine diseases in the US, and ultimately, provided a substantial support to the mitigation of disease impact in the country. The evolution of analytical capabilities for big data analysis is expected to result in novel and innovative tools that will allow for the routine analysis of big datasets, such as that collected in the MSHMP. The promotion of social initiatives, intended to promote data sharing and self-organization of producers, along with the application of those novel analytical techniques, may help to reshape in the near future the landscape of coordinated activities to promote the prevention and control of food animal diseases worldwide.

## Data Availability

No datasets were generated or analyzed for this study.

## Author Contributions

All authors listed have made a substantial, direct and intellectual contribution to the work, and approved it for publication.

### Conflict of Interest Statement

The authors declare that the research was conducted in the absence of any commercial or financial relationships that could be construed as a potential conflict of interest.
